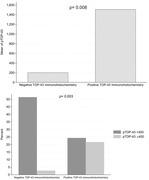# Immunoassay and Immunohistochemistry of TDP‐43 and the association with annual rate of hippocampal atrophy in Alzheimer's disease

**DOI:** 10.1002/alz.089704

**Published:** 2025-01-09

**Authors:** Hossam Youssef, Rodolfo G. Gatto, Nha Trang Thu Pham, Ronald C. Petersen, Dennis W. Dickson, Leonard Petrucelli, Jennifer L. Whitwell, Mercedes Prudencio, Keith A. Josephs

**Affiliations:** ^1^ Mayo Clinic, Rochester, MN USA; ^2^ Mayo Clinic, ROCHESTER, MN USA; ^3^ Mayo Clinic Alzheimer's Disease Research Center, Rochester, MN USA; ^4^ U.S. Advisory Council on Alzheimer’s Research, Care, and Services, Washington, DC USA; ^5^ Department of Neurology, Mayo Clinic, Rochester, MN USA; ^6^ Department of Neuroscience, Mayo Clinic, Jacksonville, FL USA; ^7^ Mayo Clinic, Jacksonville, FL USA; ^8^ Department of Radiology, Mayo Clinic, Rochester, MN USA

## Abstract

**Background:**

Transactive response DNA‐binding protein of 43 kDa (TDP‐43) is implicated in numerous neurodegenerative diseases, including Alzheimer’s disease (AD). Immunohistochemically stained TDP‐43 is linked to faster rates of hippocampal atrophy (HA) in AD. However, it is unclear whether phosphorylated TDP‐43 (pTDP‐43) measured using an immunoassay is also associated with HA. We explored associations between pTDP‐43 levels, TDP‐43 immunohistochemistry, and annual rate of HA in AD. We hypothesized that higher pTDP‐43 levels would be associated with positive TDP‐43 immunohistochemistry, and faster rates of HA.

**Method:**

Thirty‐seven cognitively impaired participants from the Mayo Clinic Alzheimer’s Disease Research Center underwent serial brain MRIs and brain autopsy. Hippocampal TDP‐43 was detected histochemically, and pTDP‐43 was quantified using biochemical assay. Hippocampal volumes were measured using the longitudinal pipeline in FreeSurfer‐7 and annualized HA rates were calculated. Linear regression analyzed the relationship between HA and pTDP‐43 biochemical assay levels. pTDP‐43 was categorized as positive (≥400) or negative (<400), and logistic regression tested the association of these levels with HA rates. The correlation between TDP‐43 immunohistochemistry status and biochemical assay levels was evaluated using descriptive statistics. All analyses were adjusted for potential confounders.

**Results:**

The mean age at death was 83.5 (±9.9) years with 49% females. Median scan interval was 3‐years. A negative relationship was observed between age at death and annual HA rate (β=‐3.1; 95% CI [‐5.3,‐0.9]; p=0.007), and a positive relationship was observed between pTDP‐43 level and annual HA rate (β=0.02; 95% CI [0.003,0.03]; p=0.015). A one‐unit increase in HA rate was associated with a 3% increase in the odds of being pTDP‐43 positive (≥400) (odds ratio=1.03; CI [1.007,1.05]; p=0.014). Cases that tested positive for Immunohistochemistry TDP‐43 had significantly higher mean pTDP‐43 immunoassay levels (1508 ± 2072 vs. 202 ± 131; p=0.008). More cases with positive IHC TDP‐43 surpassed the pTDP‐43 immunoassay cut‐off level of 400 vs the negative participants (p=0.003).

**Conclusion:**

Higher pTDP‐43 levels in the hippocampus are linked to faster HA in AD and correlated with immunohistochemistry TDP‐43 positivity. These findings underscore the role of TDP‐43 in AD neurodegeneration. The biological and clinical implications of these observations necessitate further exploration in subsequent studies.